# Myostatin in idiopathic inflammatory myopathies: Serum assessment and disease activity

**DOI:** 10.1111/nan.12849

**Published:** 2022-10-07

**Authors:** Alexandrine Mahoudeau, Céline Anquetil, Nozomu Tawara, Hossein Khademian, Damien Amelin, Loïs Bolko, Marco Silvestro, Julian Dal Cin, Bérénice Tendrel, Virginie Tardif, Kubéraka Mariampillai, Gillian Butler‐Browne, Olivier Benveniste, Yves Allenbach

**Affiliations:** ^1^ INSERM, Center of Research in Myology UMRS 974 Sorbonne Université Paris France; ^2^ Department of Internal Medicine and Clinical Immunology Sorbonne Université, Pitié‐Salpêtrière Hospital Paris France; ^3^ INSERM, Association Institut de Myologie, Center of Research in Myology UMRS 974 Sorbonne Université Paris France

**Keywords:** biomarker, disease activity, idiopathic inflammatory myopathies, muscle, myostatin

## Abstract

**Aims:**

In idiopathic inflammatory myopathies (IIM), disease activity is difficult to assess, and IIM may induce severe muscle damage, especially in immune‐mediated necrotising myopathies (IMNM) and inclusion body myositis (IBM). We hypothesise that myostatin, a negative regulator of muscle mass, could be a new biomarker of disease activity and/or muscle damage.

**Methods:**

Prospective assessment of myostatin protein level in 447 IIM serum samples (dermatomyositis [DM], *n* = 157; IBM, *n* = 72; IMNM, *n* = 125; and antisynthetase syndrome [ASyS], *n* = 93) and 59 healthy donors (HD) was performed by ELISA. A gene transcript analysis was also carried out on 18 IIM muscle biopsies and six controls to analyse myostatin and myostatin pathway‐related gene expression.

**Results:**

IIM patients had lower myostatin circulating protein levels and gene expression compared to HD (2379 [1490; 3678] pg/ml vs 4281 [3169; 5787] pg/ml; *p* < 0.0001 and log2FC = −1.83; *p* = 0.0005, respectively). Myostatin‐related gene expression varied accordingly. Based on the Physician Global Assessment, inactive IIM patients showed higher myostatin levels than active ones. This was the case for all IIM subgroups, except IMNM where low myostatin levels were maintained (2186 [1235; 3815] vs 2349 [1518; 3922] pg/ml; *p* = 0.4).

**Conclusions:**

Myostatin protein and RNA levels are decreased in all IIM patients, and protein levels correlate with disease activity. Inactive ASyS and DM patients have higher myostatin levels than active patients. Myostatin could be a marker of disease activity in these subgroups. However, IMNM patients do not have significant increase in myostatin levels after disease remission. This may highlight a new pathological disease mechanism in IMNM patients.

## Graphical abstract

We assessed transcriptomic myostatin levels in muscle biopsies and circulating levels in the serum of idiopathic inflammatory patients compared to healthy donors. We found out that myostatin expression is decreased and correlates with disease activity, thus rising up when patients' disease activity is controlled, particularly in dermatomyositis and antisynthetase syndrome patients. However, circulating myostatin levels did not increase in immune‐mediated necrotising myopathies patients, suggesting new disease mechanism.

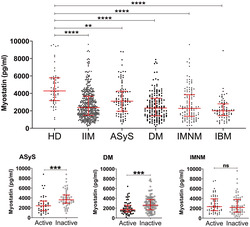

**Key points**

- Circulating myostatin level is decreased in idiopathic inflammatory myopathies.
- Myostatin pathway expression is decreased at the transcriptomic level in patients' muscle biopsies.
- Circulating myostatin levels could be a disease activity marker for antisynthetase syndrome and dermatomyositis patients.
- Circulating myostatin levels do not correlate with disease activity for immune‐mediated necrotising myopathies patients, indicating a new disease mechanism.

## INTRODUCTION

Idiopathic inflammatory myopathies (IIM) are a group of rare autoimmune diseases that can be divided into four subgroups: antisynthetase syndrome (ASyS), dermatomyositis (DM), immune‐mediated necrotising myopathy (IMNM) and inclusion body myositis (IBM) [1, 2]. These subgroups differ in terms of pathogenesis, phenotype and prognosis [3, 4, 5].

The major characteristic of IIM is muscle weakness with different degrees of severity being observed between subgroups. ASyS and DM patients have mild to moderate muscle involvement, whereas IMNM patients display the most severe muscle weakness with poor recovery of muscle strength after remission [6, 7]. Although most DM, IMNM and ASyS patients present an acute onset of symptoms, IBM is characterised by slowly progressing muscle weakness.

Muscle weakness can be a consequence of both muscle injury due to active inflammation and/or muscle damage with fibrosis, fatty replacement or muscle atrophy [8].

Currently, the International Myositis Assessment and Clinical Studies Group (IMACS) has established core set measures which allow the assessment of disease activity and damage in IIM [9]. However, no easy biomarker is available to identify and evaluate disease activity along with muscle damage. At present, muscle enzymes, such as creatine kinase (CK), are routinely used as surrogate biomarkers, and although their level correlates well with activity in IMNM [7], it lacks accuracy for the other subgroups [10].

Myostatin is a protein from the transforming growth factor beta (TGF‐β)‐1 family, mainly secreted by skeletal muscle, which acts as a negative regulator of muscle mass [11, 12]. It has already been shown to be correlated with clinical evaluation of disease activity in IIM [13] and decreased in muscle‐wasting diseases [14].

In this study, our aim is to first determine whether the myostatin level could be an appropriate marker to assess disease activity and second, if it can be used to distinguish disease activity from muscle damage.

## MATERIAL AND METHODS

### Patients

A total of 300 patients with IIM and 59 healthy donors (HD) were enrolled in the study. IIM patients were prospectively enrolled between 2013 and 2019 in a tertiary centre of IIM (Pitié Salpêtrière Hospital, Paris, France). They fulfilled the American College of Rheumatology/European League Against Rheumatism classification criteria for myositis [15]. Patients were classified into the four IIM categories: IBM patients following Lloyd's criteria [3], IMNM patients following the European Neuromuscular Centre criteria [4], DM following the European Neuromuscular Centre criteria [5] and ASyS patients based on the presence of antisynthetase autoantibodies.

Sera were collected at diagnosis or during follow‐up and kept in our biobank MASC (Myositis, DNA, Serum, Cells, ClinicalTrials.gov Identifier: NCT04637672). Ninety‐six patients had two or more serum samples at different time points, giving a total of 447 samples for the 300 patients.

HD samples were obtained from a French blood bank (Etablissement Français du Sang ref: C CPSL UNT‐N°18/EFS/033).

### Disease activity

IMACS core set measures, Manual Muscle Testing 8 (MMT8) and Physician Global Assessment (PGA) were assessed [16]. Disease activity was evaluated at the time of blood collection, and the results were represented on a numeric scale (from 0 to 10; 0 corresponding to remission without treatment and 10 the maximum disease activity). We arbitrarily defined patients as active when their PGA > 5. The PGA assessment was performed by two expert physicians independently on 40 patients with a 92.1% concordance. The rest of the cohort was assessed by one or the other.

### Myostatin, creatinine and creatine kinase measurements

Sera were quickly frozen after centrifugation and stored at −80°C until analysed. Samples were thawed only once to avoid freeze and thaw cycles. Myostatin serum levels were assessed using a commercially available ELISA kit (R&D Systems DGDF80, Minneapolis, USA) following the manufacturer's instructions. The absorbance was measured at 450 nm using a Spark plate reader (Tecan, Switzerland).

Creatinine and creatine kinase measurements were performed at the time of blood collection for IIM patients by the clinical biochemical laboratory as a routine procedure at the Pitié Salpêtrière Hospital. For HD, creatinine was measured at the time of serum myostatin on thawed serum samples. One patient with kidney dysfunction was excluded from the creatinine analysis (*n* = 1).

### Transcriptomic analysis of the myostatin pathway signature

Twenty‐four muscle samples were collected for diagnostic purposes: 18 IIM patients (six DM, six IMNM and six IBM), and six patients with normal muscle histopathology. Muscle samples were frozen in pre‐cooled isopentane. RNA was extracted from 20 frozen sections (20 μm) using QIAzol Lysis reagent and RNeasy Plus Universal Mini Kit (Qiagen, Germany) following the manufacturer's protocol. Non‐strand‐oriented libraries were prepared following the NEBNEXT single cell/low input RNA library prep kit protocol from NEB (New England Biolabs, Ipswich, MA, USA), starting from 20 ng of high‐quality total RNA. Paired‐end (2 × 75 bp) sequencing was performed on an Illumina Nextseq 500 platform.

A myostatin pathway signature was defined based on articles and reviews [17, 18, 19, 20]. We looked at the myostatin gene (MSTN) along with genes that can be (1) regulated by myostatin (*MYOG: myogenin* and *MYF5: myogenic factor 5*, two genes involved in the myogenesis and downregulated by myostatin, or *TRIM63: Tripartite Motif Containing 63* and *FBXO32: F‐box Protein 32*, two atrogenes upregulated by myostatin) or (2) regulators of myostatin (*FST: follistatin, INHBA: inhibin subunit beta A* and *DCN: decorin*).

### Statistical analyses

Data are expressed as median with the interquartile range. The Mann–Whitney test was used to compare two groups. Three or more groups were compared using the Kruskal–Wallis test followed by Dunn's multiple comparisons test. Correlation analyses were done using Spearman's rank correlation coefficient. For correlations, *r* between 0.2 and 0.39 was considered a weak correlation, *r* between 0.4 and 0.59 a moderate one and *r* more than 0.6 a strong one. The *p* < 0.05 was considered statistically significant. Statistical analyses were done using GraphPad Prism 8 for Windows (GraphPad Software, San Diego, California USA).

Multivariate linear regression analyses were performed to determine the association of myostatin, CK levels, creatinine, age or treatment (binary outcome: treated or non‐treated) with MMT8 or PGA at the first time point of blood sampling. Myostatin, creatinine and CK values were standardised prior to analysis. Data were then analysed using R program version 4.0.2 and are expressed as estimate [IC 95%].

RNA‐sequencing analyses were performed using STAR program version 2.7.1a for sequence alignment. Data were then analysed using R program version 4.0.2 and are expressed as log2 fold change (Log2FC), adjusted *p*‐value. A cut‐off at *p* < 0.05 was applied. Data are represented using GraphPad Prism 8 for Windows (GraphPad Software, San Diego, California USA).

## RESULTS

### Concentration of myostatin in the serum of IIM patients vs controls

**FIGURE 1:**
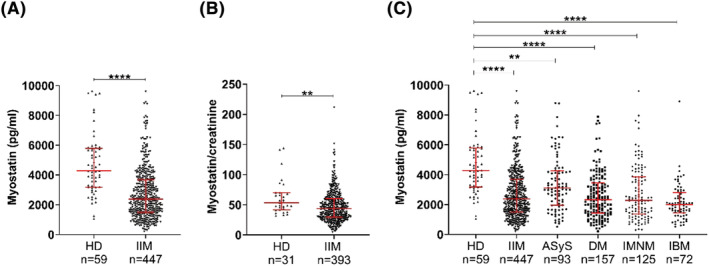
Quantification of circulating myostatin in IIM compared to HD. Circulating myostatin levels measured in HD and IIM patients sera (A), normalisation of myostatin to creatinine levels (B) and circulating myostatin levels in the four IIM patients subgroups sera (C). HD, healthy donors; IIM, idiopathic inflammatory myopathies; ASyS, antisynthetase syndrome; IMNM, immune‐mediated necrotising myopathy; DM, dermatomyositis; IBM, inclusion body myositis

Age, sex and clinical characteristics of each subgroup and HD are provided in Table 1. Myostatin was assessed in the 447 serum samples from 300 IIM patients and compared to 59 HD. At the protein level, the amount of myostatin was significantly decreased in IIM patients compared to HD (2379 [1490; 3678] pg/ml vs 4281 [3169; 5787] pg/ml; *p* < 0.0001) (Figure 1A). It should be noted that patients were slightly younger in the HD group compared to IIM (age: 45 [34; 54] vs 56 [42; 66.75]; *p* < 0.0001) and the sex ratio was different (sex ratio male 32% IIM vs 69% HD; *p* < 0.0001). We observed that myostatin did not correlate with age in IIM (*r* = −0.03; *p* = 0.49) (Supporting Information Figure S1A). There was, however, a weak positive correlation between myostatin and age for HD (Supporting Information Figure S1B). Our IIM patients were slightly older than HD, but myostatin levels were decreased. In IIM, myostatin levels were slightly decreased in women compared to men (2192 [1367;3440] pg/ml vs 2658 [1664;3967] pg/ml; *p* = 0.0175) (Supporting Information Figure S1C). There was, however, no difference between men and women in the HD population (Supporting Information Figure S1D).

**TABLE 1:** Patients' and HD characteristics at the first time‐point of blood sampling

	ASyS	IMNM	DM	IBM	HD
*N*	64	85	87	64	59
Age (year)	48 [34.25; 60]	51 [35.5; 63]	53 [42; 66]	65.5 [59; 74]	45 [34; 54]
Sex M/F	20/44	23/62	20/67	32/32	41/18
Treatment GC (treated/non‐treated)	38/24	47/32	56/21	4/45	NA
Dose GC (mg/day)	15 [5.5; 40]	7.5 [5; 20]	15 [7; 42.5]	8 [5.5; 17.25]	NA
CK (UI/L)	394 [126.3; 1483]*n* = 60	908 [310.5; 3311]*n* = 81	126 [55.75; 380]*n* = 82	408.5 [263.3; 661]*n* = 60	NA
PGA (min 0–max 10)	6 [3; 7]*n* = 61	6 [5; 7]*n* = 83	6 [3; 7]*n* = 87	ND	NA
MMT8 (min 0–max 150)	150 [142; 150]*n* = 59	141 [122; 148]*n* = 77	142 [130; 150]*n* = 76	138 [131.5; 146]*n* = 53	NA
Creatinine (μmol/L)	62 [49; 71]*n* = 55	46 [35; 55]*n* = 74	56.5 [44.25; 68.75]*n* = 80	51 [39; 66]*n* = 53	84 [71; 89]*n* = 31

Abbreviations: GC, glucocorticoids; M/F, male/female; CK, creatine kinase; MMT8, manual muscle testing 8; PGA, physician global assessment; ASyS, anti‐synthetase syndrome; IMNM, immune‐mediated necrotising myopathy; DM, dermatomyositis; IBM, inclusion body myositis; HD, healthy donors; ND, not done; NA, not available.

Because myostatin is a protein mainly secreted by skeletal muscle [11, 12], in order to confirm that this decrease in myostatin did not reflect a difference in muscle mass due to other factors such as age, sex and/or physical training of the patients, the results were normalised to creatinine. In healthy individuals with stable kidney function, serum creatinine levels correlate with muscle mass [21, 22, 23, 24]. Thus, it was used in our study as a surrogate marker of muscle mass. A moderate correlation was found between myostatin and serum creatinine levels (*r* = 0.51; *p* < 0.0001; data not shown). After normalisation, the level of myostatin was still statistically significantly lower in IIM patients compared to HD (43.73 [29.25; 60.24] vs 53.51 [41.64; 69.97]; *p* = 0.002) (Figure 1B).

The level of myostatin was also assessed in each of the four myositis subgroups and again compared to HD (ASyS *n* = 93, DM *n* = 157, IMNM *n* = 125 and IBM *n* = 72). Myostatin levels were lower in ASyS (3109 [1952; 4258] pg/ml; *p* = 0.001), DM (2327 [1431; 3446] pg/ml; *p* < 0.0001), IMNM (2285 [1371; 3851] pg/ml; *p* < 0.0001) and IBM (2005 [1449; 2803] pg/ml; *p* < 0.0001) compared to HD (4281 [3169; 5787] pg/ml) (Figure 1C).

### Transcriptomic analysis of the myostatin pathway

**FIGURE 2:**
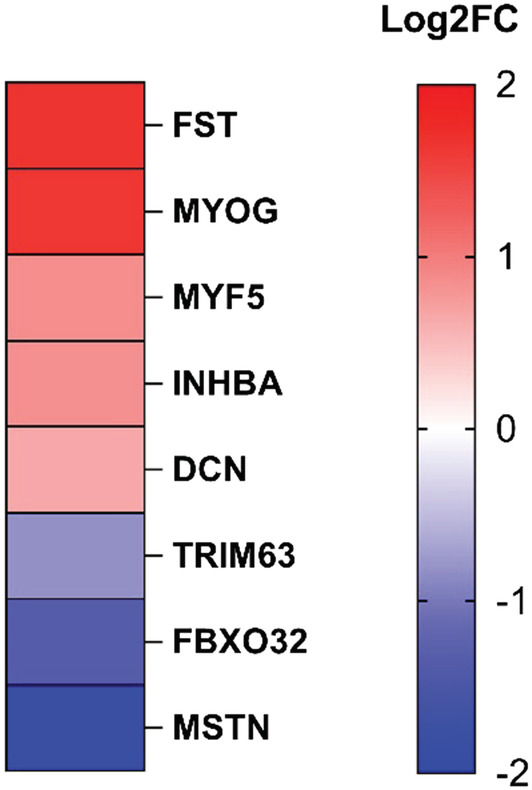
Myostatin gene pathway expression in IIM vs HD. Transcriptomic analysis of myostatin pathway when comparing 18 IIM muscle biopsies (six DM, six IBM and six IMNM) vs six controls. Data are expressed as log2FC. FST, follistatin; MYOG, myogenin; MYF5, myogenic factor 5; INHBA, inhibin subunit beta A; DCN, decorin; TRIM63, tripartite motif containing 63; FBXO32, F‐box protein 32; MSTN, myostatin

Because myostatin is mainly produced by skeletal muscle, the decrease in blood might be consequent to a drop in myostatin production and subsequently be linked to the downregulation of pathways related to its expression in muscle tissue. In order to test this hypothesis, transcriptomic analysis was carried out on muscle biopsies from 18 IIM patients (six DM, six IMNM and six IBM) and six healthy controls (Figure 2). Analysis of the differentially expressed genes showed downregulation of *myostatin* (*MSTN*) in all IIM patient biopsies compared to healthy controls (log2FC = −1.83; *p* = 0.0005). The expression of several genes, related to the myostatin pathway varied in accordance with this down‐regulation. We observed an upregulation of the myogenesis genes, *myogenin* (*MYOG*) (log2FC = 1.62; *p* < 0.0001) and *myogenic factor 5* (*MYF5*) (log2FC = 0.86; *p* = 0.04), normally downregulated by myostatin. We also show a downregulation of the atrophy genes *F‐box only protein 32* (*FBXO32*) (log2FC = −1.35; *p* < 0.0001) coding for the protein atrogin, along with *E3 ubiquitin‐protein ligase TRIM63* (log2FC = −0.82; *p* = 0.02) coding for the protein MuRF1. These two genes are usually activated by myostatin. Moreover, *follistatin* (*FST*) (log2FC = 1.66; *p* < 0.0001), *inhibin subunit beta A* (*INHBA*) (log2FC = 0.84; *p* = 0.04) coding the activin A subunit inhibin βA and *decorin* (*DCN*) (log2FC = 0.65; *p* = 0.03), all of which are inhibitors or agonists of myostatin, were found to be upregulated.

Among the 18 patients used for the muscle biopsy study, seven had myostatin measurements at the same time: two DM, two IBM and three IMNM. Circulating myostatin levels in these seven patients were significantly decreased compared to HD (1539 [800.7;3877] pg/ml vs 4281 [3169;5787] pg/ml; *p* < 0.005; data not shown).

Altogether, these results support a downregulation of the myostatin pathway in the muscle, which shows that the decreased level of myostatin detected in the serum mirrors its activity within the muscle compartment.

### Myostatin and IIM disease activity

Based on the Physician Global Assessment, IIM patients were divided into active and inactive patients. IBM patients were excluded from this analysis because their PGA cannot be established.

**FIGURE 3:**
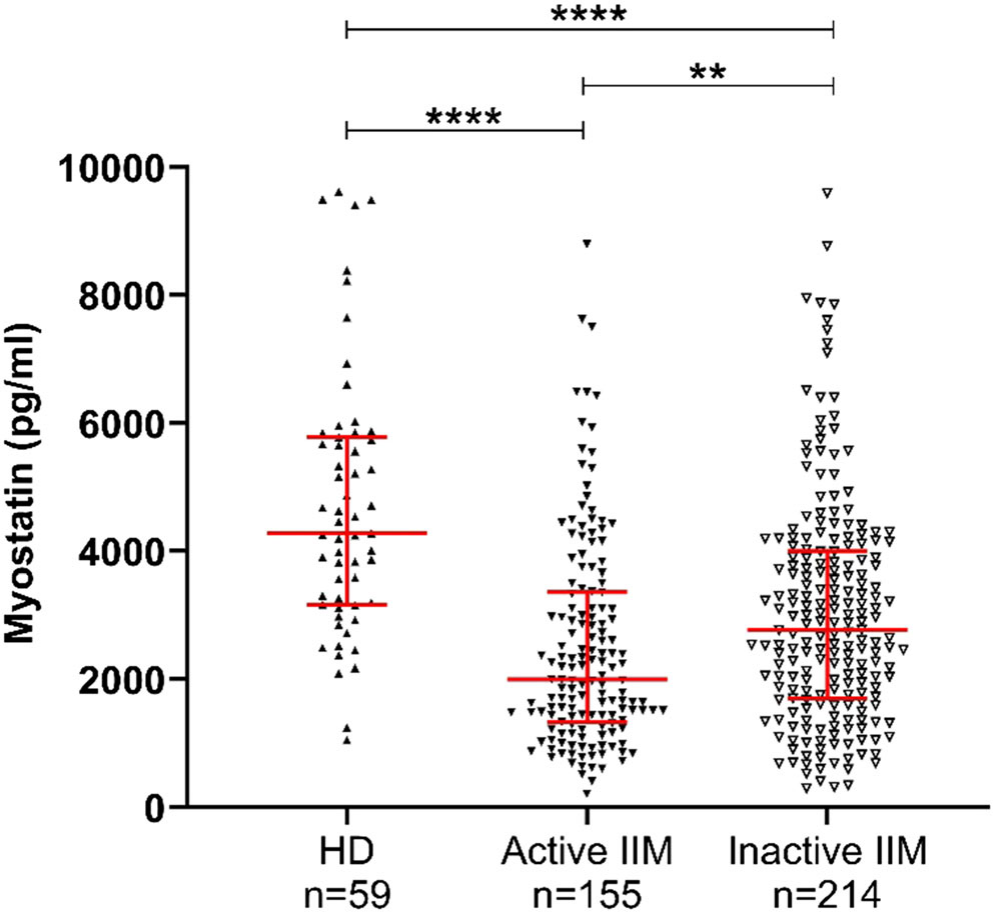
Myostatin levels in active and inactive IIM patients compared to HD. Circulating myostatin levels measured in HD and in inactive or active IIM patients. Patients with a PGA > 5 were considered active. HD, healthy donors; IIM, idiopathic inflammatory myopathies. **p < 0.01; ****p < 0.0001

Inactive IIM patients were found to have higher myostatin levels compared to active ones (2778 [1701; 4005] pg/ml vs 2003 [1338; 3366] pg/ml; *p* = 0.006) but were consistently lower than HD myostatin levels (4281 [3169; 5787] pg/ml; *p* < 0.0001) (Figure 3).

The myostatin gene promoter contains glucocorticoid response elements that can induce myostatin expression [25, 26]. Disease control in inactive patients may have been achieved using glucocorticoids. Thus, we wanted to check if there was a correlation between myostatin levels and glucocorticoid doses. No correlation between the two was observed (*r* = −0.1883 *p* = 0.0032) (Supporting Information Figure S2).

**FIGURE 4:**
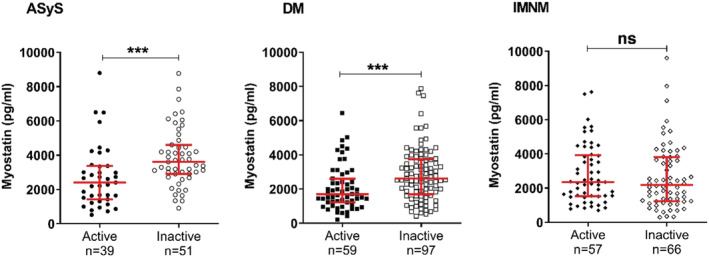
Myostatin levels in active vs inactive IIM patients. Circulating myostatin level measured in inactive or active IIM patients subgroups. Patients with a PGA > 5 were considered active. ASyS, anti‐synthetase syndrome; DM, dermatomyositis; IMNM, immune‐mediated necrotising myopathy; ns, non‐significant. *** *p* < 0.001

The comparison between subgroups showed that ASyS and DM inactive patients had higher myostatin levels compared to their active counterparts (3611 [2910; 4603] pg/ml vs 2407 [1420; 3366] pg/ml; *p* = 0.0004, and 2618 [1690; 3749] pg/ml vs 1697 [1227; 2606] pg/ml; *p* = 0.0005, respectively) (Figure 4). ASyS inactive patients even reached the myostatin level of HD (3611 [2910; 4603] pg/ml vs 4281 [3169; 5787] pg/ml; *p* = 0.54). However, the myostatin levels in IMNM patients did not show any difference between inactive and active patients (2186 [1235; 3815] vs 2349 [1518; 3922] pg/ml; *p* = 0.4) (Figure 4).

Following this notable difference in the IMNM subgroups, we wondered if there would be a difference between IMNM patients with anti‐SRP or anti‐HMGCR autoantibodies. We only had eight seronegative patients in our cohort so we did not include these patients in the analysis. We did not find a difference between anti‐SRP and anti‐HMGCR patients (Supporting Information Figure S3A) nor between active and inactive patients (Supporting Information Figure S3B).

Following univariate analysis, the PGA and myostatin levels showed a weak negative correlation in all IIM patients (*r* = −0.28; *p* < 0.0001) (Table 2). By subgroup analysis, a moderate negative correlation was found between myostatin and the PGA for ASyS (*r* = −0.40; *p* < 0.0001) and DM patients (*r* = −0.46; *p* < 0.0001) but no correlation for IMNM patients (*r* = 0.0009; *p* = 0.99). The MMT8 and myostatin levels had a moderate positive correlation (*r* = 0.47; *p* < 0.0001) in IIM patients. By subgroups, there was a weak positive correlation between myostatin and the MMT8 for ASyS (*r* = 0.35; *p* = 0.0009) and IBM (*r* = 0.32; *p* = 0.01) patients, and a moderate positive one for DM (*r* = 0.5; *p* < 0.0001) and IMNM patients (*r* = 0.43; *p* < 0.0001).

**TABLE 2:** Myostatin association with clinical parameters in IIM patients

		IIM	ASyS	DM	IMNM	IBM
Myostatin	PGA	*r* = −0.28*p* < 0.0001	*r* = −0.40*p* < 0.0001	*r* = −0.46*p* < 0.0001	*r* = 0.0009*p* = 0.99	NA
	MMT8	*r* = 0.47*p* < 0.0001	*r* = 0.35*p* = 0.0009	*r* = 0.5*p* < 0.0001	*r* = 0.43*p* < 0.0001	*r* = 0.32*p* = 0.01

*Note*: Univariate correlation analysis between myostatin and PGA or MMT8 in IIM and IIM subgroups.

Abbreviations: MMT8, manual muscle testing of 8 muscles; PGA, physician global assessment; ASyS, antisynthetase syndrome; IIM, idiopathic inflammatory myopathies; IMNM, immune‐mediated necrotising myopathy; DM, dermatomyositis; IBM, inclusion body myositis.

Several predictive factors were used in a linear multivariate regression model: CK level, myostatin levels, creatinine levels, treatment and age at the time of the first sample. For all IIM patients, CK (0.77 [0.53; 1.02]; *p* < 0.0001) and, to a lesser extent, myostatin (−0.46 [−0.77; −0.15]; *p* = 0.003) and age (0.02 [0.004; 0.04]; *p* = 0.01) were independent factors associated with disease activity. Myostatin (2.91 [0.32; 5.5]; *p* = 0.03) and creatinine (7.82 [5.26; 10.39]; *p* < 0.0001) were the two independent factors associated with MMT8 scores (Table 3). Only myostatin was a significant independent factor in both models.

**TABLE 3:** Association of CK levels, myostatin, creatinine, age or glucocorticoid treatment with MMT8 or PGA in IIM patients

	Myostatin	Creatinine	CK	Age	Treatment
**PGA**	−0.46 [−0.77;‐0.15]*p* = 0.003	−0.08 [−0.4;0.23]*p* = 0.6	0.77 [0.53;1.02]*p* < 0.0001	0.02 [0.004;0.04]*p* = 0.01	−0.53 [−1.08;0.03]*p* = 0.06
**MMT8**	2.91 [0.32;5.5]*p* = 0.03	7.82 [5.26;10.39]*p* < 0.0001	−1.42 [−3.70;0.87]*p* = 0.22	−0.12 [−0.25;0.007]*p* = 0.06	−0.47 [−4.8;3.87]*p* = 0.83

*Note*: Multivariate analysis estimating the association of CK levels, myostatin, creatinine, age or glucocorticoid treatment (binary outcome: treated or non‐treated) with MMT8 or PGA in all IIM patients at their first time‐point of blood sampling.

Abbreviations: CK, creatine kinase; MMT8, manual muscle testing of 8 muscles; PGA, physician global assessment.

## DISCUSSION

In this study, the level of myostatin was assessed in a large and well‐characterised cohort of IIM patients. We showed that myostatin was decreased at the protein level in serum and at the transcriptomic level in muscle biopsies of all IIM patients. Myostatin was also correlated with the PGA and the MMT8. Moreover, myostatin level was higher in inactive IIM patients compared to active ones, except for IMNM patients where it remained unchanged.

Only a few studies have previously examined myostatin in IIM and they are often contradictory [13, 14, 27, 28, 29, 30]. Some results are concordant with our studies [13, 14], whereas others are not [27, 28, 29, 30], but the comparison is difficult because there are significant variations in the studied populations (patients number, clinical status and IIM classification).

Among our study's strengths, (i) we had the chance to analyse the largest cohort of patients, (ii) that allowed us to classify patients using the most recent IIM subclassification (four subgroups), and (iii) we correlated the disease activity with myostatin levels.

Only two studies have enrolled more than one IIM subgroup. The first one included Polymyositis (PM), DM and IMNM patients and showed decreased circulating myostatin levels, as we observed [13]. They, however, did not observe a decrease at the transcriptomic level but only a dysregulation of the myostatin pathway in general. In the second study, circulating myostatin levels were the same in IIM subgroups (DM and PM) compared to controls [27].

Concerning IIM subgroups, one study found a borderline decrease of myostatin in IBM serum [14]. That might be due to the small number of controls. The decrease was, however, significant at the transcriptomic level in muscle biopsies, as we observed. Another study found an increase of myostatin protein in IBM muscle, but it was trapped in the Aβ peptide aggregates as shown also in another study [29]. For DM patients, a tendency for a decrease in myostatin in the serum was observed, as we demonstrated [13]. However, one other study found upregulation in DM, whereas De Sordi et al found no difference at all [27, 28]. It should be noted that they studied inactive DM patients, whereas we enrolled a high proportion of DM patients with active disease. Regarding IMNM, Vernerová et al observed a tendency to a decrease in circulating myostatin levels, in line with our results [13]. Finally, in PM, circulating myostatin levels were also observed to be decreased in two studies [13, 27]. PM is a heterogenous subgroup composed of both IMNM and ASyS patients, and, consequently, we were not able to compare these results with our study.

The differences in circulating myostatin levels in IIM patients compared to controls and in active compared to inactive patients could have several explanations. We first looked at the effect of glucocorticoid treatment because myostatin might be induced by glucocorticoids [25, 26]. However, we did not see any effect of glucocorticoid treatment on myostatin levels, in line with previous studies [13, 27].

Myostatin levels could also depend on muscle mass. This is why we normalised myostatin levels to creatinine levels, as creatinine has been proposed to be a biomarker of muscle mass [21, 22, 23, 24]. Circulating myostatin levels were still decreased even after this normalisation. However, creatinine normalisation has some limitations, as it is also a biomarker of kidney clearance and function. Other studies have normalised circulating myostatin levels to either the body mass index or dual‐energy x‐ray absorptiometry results, but using this approach, no significant association between myostatin levels and lean muscle mass was observed [13, 27]. The association of myostatin and muscle mass has not yet been clearly demonstrated, probably because muscle mass is difficult to assess. Sex and age correlations with myostatin levels are also still debated because they may also influence muscle mass [31].

Myostatin levels can be modified by inflammation. The decrease in circulating myostatin levels we observed could be due to an increase in follistatin, antagonising its action [32]. Follistatin can be upregulated during inflammation by activin as well as by other cytokines, such as IL1β, TNFα and IFNγ [19]. These cytokines are indeed increased in IIM, especially during the active phase of the disease [33, 34], suggesting a link between myostatin level and disease activity.

Interestingly, in our study, IMNM patients showed a distinct profile with the same circulating myostatin levels in patients with active or inactive disease. IMNM patients have the most severe muscle damage and the worst recovery among the IIM, with only half of them recovering after treatment [6, 35, 36]. This difference with the other IIMs, as discussed above, could be due to a reduced muscle mass due to fibro‐fatty replacement, but we cannot rule out a persistent low disease activity.

There is a critical need to distinguish disease activity from muscle damage, and myostatin individual variations would have to be studied in more depth for this purpose.

Myostatin has already been used to monitor disease progression and treatment efficacy in Duchenne muscular dystrophy and Myotubular myopathy [37, 38]. Myostatin was decreased at baseline in these two diseases and increased in treated animal models [37, 38].

Our study has limitations. We are limited by the retrospective aspect of our study, especially with no sampling calendar and no systematic follow‐up. We have also only used the PGA, MMT8 and CK levels to assess disease activity and not all core set measures of IMACS. The PGA in particular can be subjective, but we made sure of the concordance between different assessors and selected a cut‐off at 5 to make sure the disease was active.

## CONCLUSION

To conclude, in a large cohort of IIM patients, this study shows a decrease in the level of circulating myostatin and muscle myostatin RNA. Circulating myostatin levels are lower in active ASyS and DM patients and increase when the disease is controlled. Myostatin could therefore be used as a biomarker of disease activity in these subgroups. However, in IMNM patients, myostatin levels did not change with disease activity, suggesting specific underlying pathomechanisms in IMNM explaining the poor outcome of these patients. Further prospective studies are needed to validate the usefulness of myostatin in the follow‐up of myositis patients.

## CONFLICT OF INTEREST

The authors declare no conflict of interest.

## ETHICS STATEMENT

Written informed consent from each patient and approval by the local Ethics Committee (CPP Ile de France VI [2013‐12‐19]), by the Ministry of Research (CCTIRS N°14.323) and from the National Commission on Informatics and Liberties (AR158656) were obtained.

We also received research authorisation for the database from the National Commission on Informatics and Liberties (authorisation number 915139).

## AUTHOR CONTRIBUTIONS

AM, YA and OB contributed to the study design; AM did the ELISA experiments; AM, CA and HK performed data analysis; CA performed the RNA bulk experiment assay; DA, CA, NT and AM contributed to the samples banking; LB, MS and AM retrieved patients information from their clinical files; AM, YA and OB drafted the manuscript; all authors revised and approved the final version of the manuscript.

### PEER REVIEW

The peer review history for this article is available at https://publons.com/publon/10.1111/nan.12849.

## Supporting information


**Figure S1:** Effects of age and sex on myostatin levels
Correlation between circulating myostatin levels and age for IIM patients (A) and HD (B) and myostatin circulating levels difference between female and men for IIM (C) and HD (D). *p < 0.05

**Figure S2:** Myostatin and glucocorticoids levels correlation
Correlation between circulating myostatin levels and glucocorticoids for IIM patients.

**Figure S3:** Myostatin circulating levels in anti‐SRP and anti‐HMGCR IMNM patients
Circulating myostatin levels in IMNM patients with anti‐SRP or anti‐HMGCR autoantibodies compared to controls (A) and in active or inactive patients (B). Patients with a PGA > 5 were considered active.
****p < 0.0001

## Data Availability

The data that support the findings of this study are available from the corresponding author upon reasonable request.

## References

[1] Mariampillai K , Granger B , Amelin D , et al. Development of a new classification system for idiopathic inflammatory myopathies based on clinical manifestations and myositis‐specific autoantibodies. JAMA Neurol. 2018;75(12):1528‐1537. doi:10.1001/jamaneurol.2018.2598 30208379 PMC6583199

[2] Pinal‐Fernandez I , Casal‐Dominguez M , Derfoul A , et al. Machine learning algorithms reveal unique gene expression profiles in muscle biopsies from patients with different types of myositis. Ann Rheum Dis. 2020;79(9):1234‐1242. doi:10.1136/annrheumdis-2019-216599 32546599 PMC10461844

[3] Lloyd TE , Mammen AL , Amato AA , Weiss MD , Needham M , Greenberg SA . Evaluation and construction of diagnostic criteria for inclusion body myositis. Neurology. 2014;83(5):426‐433. doi:10.1212/WNL.0000000000000642 24975859 PMC4132572

[4] Allenbach Y , Mammen AL , Benveniste O , Stenzel W . Immune‐mediated necrotizing myopathies working group. 224th ENMC International Workshop: Clinico‐sero‐pathological classification of immune‐mediated necrotizing myopathies Zandvoort, the Netherlands, 14–16 October 2016. Neuromuscul Disord. 2018;28(1):87‐99. doi:10.1016/j.nmd.2017.09.016 29221629

[5] Mammen AL , Allenbach Y , Stenzel W , et al. 239th ENMC International Workshop: classification of dermatomyositis, Amsterdam, the Netherlands, 14–16 December 2018. Neuromuscul Disord. 2020;30(1):70‐92. doi:10.1016/j.nmd.2019.10.005 31791867

[6] Tiniakou E , Pinal‐Fernandez I , Lloyd TE , et al. More severe disease and slower recovery in younger patients with anti‐3‐hydroxy‐3‐methylglutaryl‐coenzyme A reductase‐associated autoimmune myopathy. Rheumatology (Oxford). 2017;56(5):787‐794. doi:10.1093/rheumatology/kew470 28096458 PMC5850825

[7] Allenbach Y , Arouche‐Delaperche L , Preusse C , et al. Necrosis in anti‐SRP+and anti‐HMGCR+myopathies: role of autoantibodies and complement. Neurology. 2018;90(6):e507‐e517. doi:10.1212/WNL.0000000000004923 29330311

[8] Landon‐Cardinal O , Koumako C , Hardouin G , et al. Severe axial and pelvifemoral muscle damage in immune‐mediated necrotizing myopathy evaluated by whole‐body MRI. Semin Arthritis Rheum. 2020;50(6):1437‐1440. doi:10.1016/j.semarthrit.2020.02.009 32222382

[9] Rider LG , Werth VP , Huber AM , et al. Measures of adult and juvenile dermatomyositis, polymyositis, and inclusion body myositis: physician and patient/parent global activity, manual muscle testing (MMT), health assessment questionnaire (HAQ)/childhood health assessment questionnaire (C‐HAQ), childhood myositis assessment scale (CMAS), myositis disease activity assessment tool (MDAAT), disease activity score (DAS), short form 36 (SF‐36), child health questionnaire (CHQ), physician global damage, myositis damage index (MDI), quantitative muscle testing (QMT), myositis functional Index‐2 (FI‐2), myositis activities profile (MAP), inclusion body myositis functional rating scale (IBMFRS), cutaneous dermatomyositis disease area and severity index (CDASI), cutaneous assessment tool (CAT), dermatomyositis skin severity index (DSSI), skindex, and dermatology life quality index (DLQI). Arthritis Care Res (Hoboken). 2011;63(Suppl 11):S118‐S157. doi:10.1002/acr.20532 22588740 PMC3748930

[10] Mathur T , Manadan AM , Thiagarajan S , Hota B , Block JA . The utility of serum aldolase in normal creatine kinase dermatomyositis. J Clin Rheumatol. 2014;20(1):47‐48. doi:10.1097/RHU.0000000000000062 24356484

[11] McPherron AC , Lee SJ . Double muscling in cattle due to mutations in the myostatin gene. Proc Natl Acad Sci U S A. 1997;94(23):12457‐12461. doi:10.1073/pnas.94.23.12457 9356471 PMC24998

[12] Deng B , Zhang F , Wen J , et al. The function of myostatin in the regulation of fat mass in mammals. Nutr Metab (Lond). 2017;14(1):29. doi:10.1186/s12986-017-0179-1 28344633 PMC5360019

[13] Vernerová L , Horváthová V , Kropáčková T , et al. Alterations in activin A–myostatin–follistatin system associate with disease activity in inflammatory myopathies. Rheumatology. 2020;59(9):2491‐2501. doi:10.1093/rheumatology/kez651 31990347

[14] Mariot V , Joubert R , Hourdé C , et al. Downregulation of myostatin pathway in neuromuscular diseases may explain challenges of anti‐myostatin therapeutic approaches. Nat Commun. 2017;8(1):1859 doi:10.1038/s41467-017-01486-4 29192144 PMC5709430

[15] Lundberg IE , Tjärnlund A , Bottai M , et al. 2017 European League against rheumatism/American College of Rheumatology classification criteria for adult and juvenile idiopathic inflammatory myopathies and their major subgroups. Ann Rheum Dis. 2017;76(12):1955‐1964. doi:10.1136/annrheumdis-2017-211468 29079590 PMC5736307

[16] Rider LG , Aggarwal R , Machado PM , et al. Update on outcome assessment in myositis. Nat Rev Rheumatol. 2018;14(5):303‐318. doi:10.1038/nrrheum.2018.33 29651119 PMC6702032

[17] Lee SJ , McPherron AC . Regulation of myostatin activity and muscle growth. Proc Natl Acad Sci U S a. 2001;98(16):9306‐9311. doi:10.1073/pnas.151270098 11459935 PMC55416

[18] Lokireddy S , Mouly V , Butler‐Browne G , et al. Myostatin promotes the wasting of human myoblast cultures through promoting ubiquitin‐proteasome pathway‐mediated loss of sarcomeric proteins. American Journal of Physiology‐Cell Physiology. 2011;301(6):C1316‐C1324. doi:10.1152/ajpcell.00114.2011 21900687

[19] Hedger MP , Winnall WR , Phillips DJ , de Kretser DM . The regulation and functions of activin and follistatin in inflammation and immunity. In: Vitamins & Hormones. 2011;85:255‐297. doi:10.1016/B978-0-12-385961-7.00013-5. 21353885

[20] Verzola D , Barisione C , Picciotto D , Garibotto G , Koppe L . Emerging role of myostatin and its inhibition in the setting of chronic kidney disease. Kidney Int. 2019;95(3):506‐517. doi:10.1016/j.kint.2018.10.010 30598193

[21] Baxmann AC , Ahmed MS , Marques NC , et al. Influence of muscle mass and physical activity on serum and urinary creatinine and serum cystatin C. Clin J am Soc Nephrol. 2008;3(2):348‐354. doi:10.2215/CJN.02870707 18235143 PMC2390952

[22] Heymsfield SB , Arteaga C , McManus C , Smith J , Moffitt S . Measurement of muscle mass in humans: validity of the 24‐hour urinary creatinine method. Am J Clin Nutr. 1983;37(3):478‐494. doi:10.1093/ajcn/37.3.478 6829490

[23] Keshaviah PR , Nolph KD , Moore HL , et al. Lean body mass estimation by creatinine kinetics. J am Soc Nephrol. 1994;4(7):1475‐1485. doi:10.1681/ASN.V471475 8161729

[24] Patel SS , Molnar MZ , Tayek JA , et al. Serum creatinine as a marker of muscle mass in chronic kidney disease: results of a cross‐sectional study and review of literature. J Cachexia Sarcopenia Muscle. 2013;4(1):19‐29. doi:10.1007/s13539-012-0079-1 22777757 PMC3581614

[25] Ma K , Mallidis C , Artaza J , Taylor W , Gonzalez‐Cadavid N , Bhasin S . Characterization of 5′‐regulatory region of human myostatin gene: regulation by dexamethasone in vitro. Am J Physiol Endocrinol Metab. 2001;281(6):E1128‐E1136. doi:10.1152/ajpendo.2001.281.6.E1128 11701425

[26] Ma K , Mallidis C , Bhasin S , et al. Glucocorticoid‐induced skeletal muscle atrophy is associated with upregulation of myostatin gene expression. American Journal of Physiology‐Endocrinology and Metabolism. 2003;285(2):E363‐E371. doi:10.1152/ajpendo.00487.2002 12721153

[27] de Sordi CM , dos Reis‐Neto ET , Keppeke GD , Shinjo SK , Sato EI . Serum myostatin and follistatin levels in patients with dermatomyositis and polymyositis. J Clin Rheumatol. 2022;28(1):33‐37. doi:10.1097/RHU.0000000000001806 34740999

[28] Kerschan‐Schindl K , Gruther W , Föger‐Samwald U , Bangert C , Kudlacek S , Pietschmann P . Myostatin and markers of bone metabolism in dermatomyositis. BMC Musculoskelet Disord. 2021;22(1):150 doi:10.1186/s12891-021-04030-0 33546660 PMC7866468

[29] Sachdev R , Kappes‐Horn K , Paulsen L , et al. Endoplasmic reticulum stress induces myostatin high molecular weight aggregates and impairs mature myostatin secretion. Mol Neurobiol. 2018;55(11):8355‐8373. doi:10.1007/s12035-018-0997-9 29546591 PMC6153721

[30] Wójcik S , Engel WK , McFerrin J , Askanas V . Myostatin is increased and complexes with amyloid‐β within sporadic inclusion‐body myositis muscle fibers. Acta Neuropathol. 2005;110(2):173‐177. doi:10.1007/s00401-005-1035-3 15983828

[31] Baczek J , Silkiewicz M , Wojszel ZB . Myostatin as a biomarker of muscle wasting and other pathologies‐state of the art and knowledge gaps. Nutrients. 2020;12(8): doi:10.3390/nu12082401 PMC7469036 32796600

[32] Amthor H , Nicholas G , McKinnell I , et al. Follistatin complexes myostatin and antagonises myostatin‐mediated inhibition of myogenesis. Dev Biol. 2004;270(1):19‐30. doi:10.1016/j.ydbio.2004.01.046 15136138

[33] De Bleecker JL , Meire VI , Declercq W , Van Aken EH . Immunolocalization of tumor necrosis factor‐alpha and its receptors in inflammatory myopathies1. This paper was presented at the 50th annual meeting of the American Academy of Neurology, Minneapolis, MN, USA, April 1998.1. Neuromuscul Disord. 1999;9(4):239‐246. doi:10.1016/S0960-8966(98)00126-6 10399751

[34] Lundberg I , Ulfgren AK , Nyberg P , Andersson U , Klareskog L . Cytokine production in muscle tissue of patients with idiopathic inflammatory myopathies. Arthritis Rheum. 1997;40(5):865‐874. doi:10.1002/art.1780400514 9153548

[35] Pinal‐Fernandez I , Parks C , Werner JL , et al. Longitudinal course of disease in a large cohort of myositis patients with autoantibodies recognizing the signal recognition particle. Arthritis Care Res (Hoboken). 2017;69(2):263‐270. doi:10.1002/acr.22920 27111848 PMC5079847

[36] Pinal‐Fernandez I , Casal‐Dominguez M , Carrino JA , et al. Thigh muscle MRI in immune‐mediated necrotising myopathy: extensive oedema, early muscle damage and role of anti‐SRP autoantibodies as a marker of severity. Ann Rheum Dis. 2017;76(4):681‐687. doi:10.1136/annrheumdis-2016-210198 27651398 PMC6019551

[37] Mariot V , Le Guiner C , Barthélémy I , et al. Myostatin is a quantifiable biomarker for monitoring pharmaco‐gene therapy in Duchenne muscular dystrophy. Mol Ther Methods Clin Dev. 2020;18:415‐421. doi:10.1016/j.omtm.2020.06.016 32695843 PMC7363622

[38] Koch C , Buono S , Menuet A , et al. Myostatin: a circulating biomarker correlating with disease in myotubular myopathy mice and patients. Mol Ther Methods Clin Dev. 2020;17:1178‐1189. doi:10.1016/j.omtm.2020.04.022 32514412 PMC7267729

