# Supplementary figures and images for: Myostatin in idiopathic inflammatory myopathies: Serum assessment and disease activity
Source: Neuropathol Appl Neurobiol. 2022 Oct 7;49(1):e12849. doi: 10.1111/nan.12849 (PMC10092350; doi:10.1111/nan.12849)

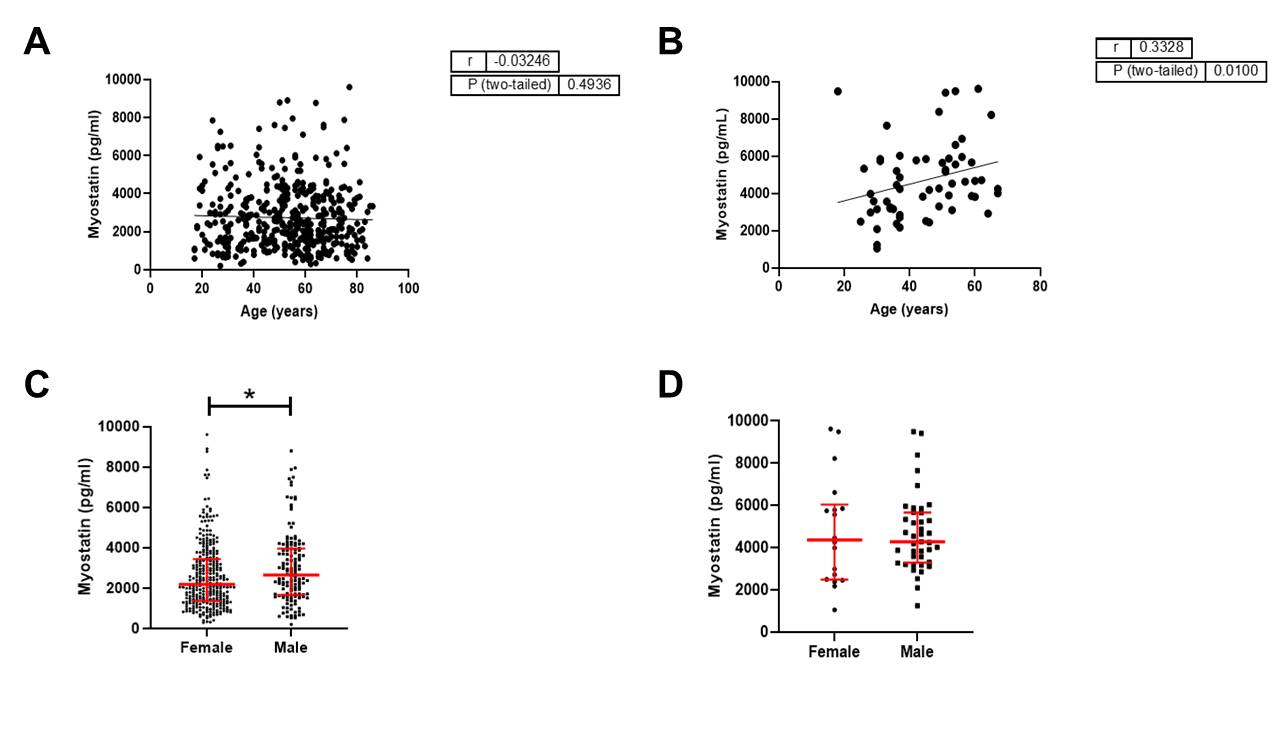

Supplement: Supplementary file 1 — Figure S1: Effects of age and sex on myostatin levels Correlation between circulating myostatin levels and age for IIM patients (A) and HD (B) and myostatin circulating levels difference between female and men for IIM (C) and HD (D). *p < 0.05 [file NAN-49-0-s002.jpg]

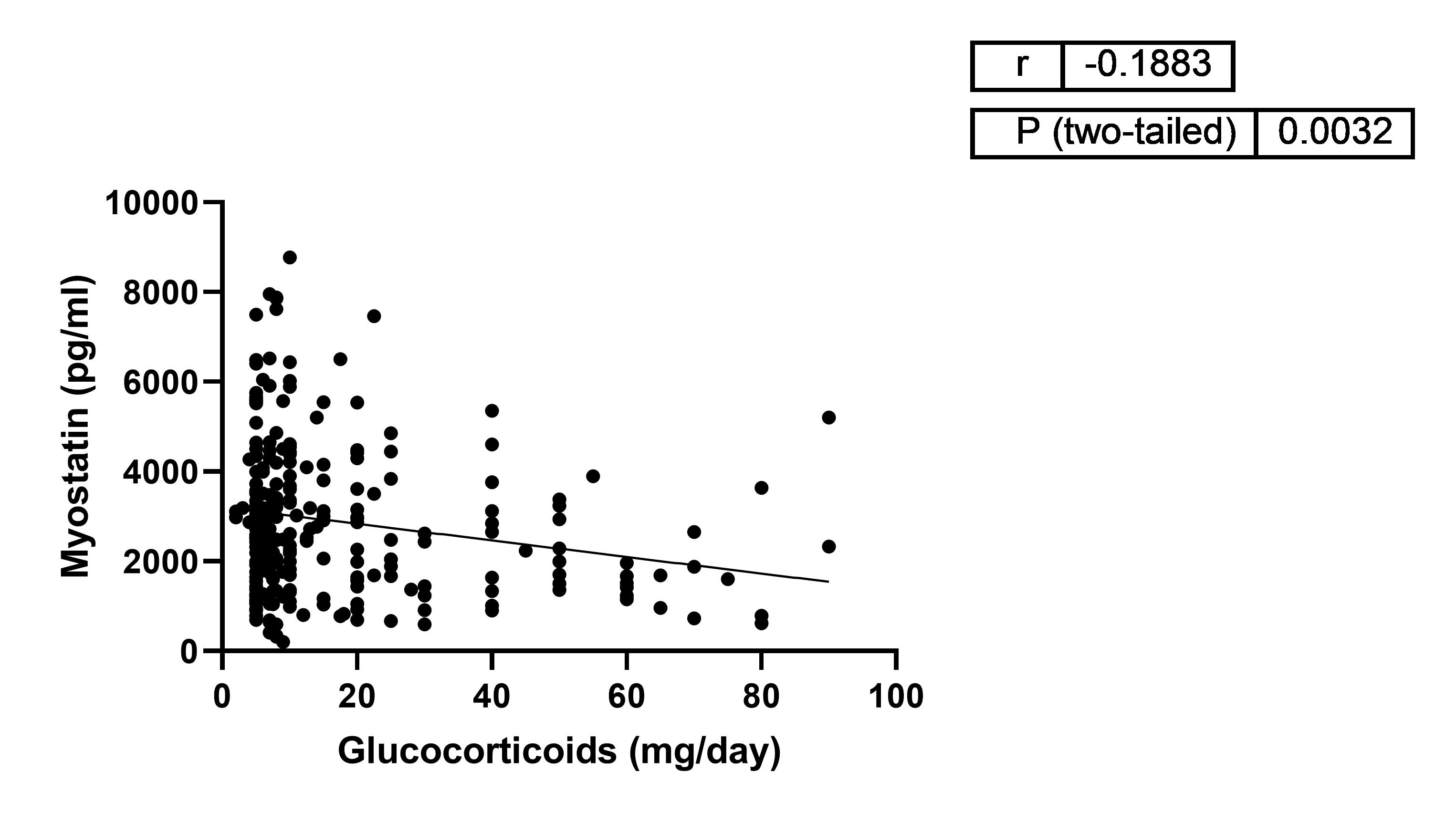

Supplement: Supplementary file 2 — Figure S2: Myostatin and glucocorticoids levels correlation Correlation between circulating myostatin levels and glucocorticoids for IIM patients. [file NAN-49-0-s001.jpg]

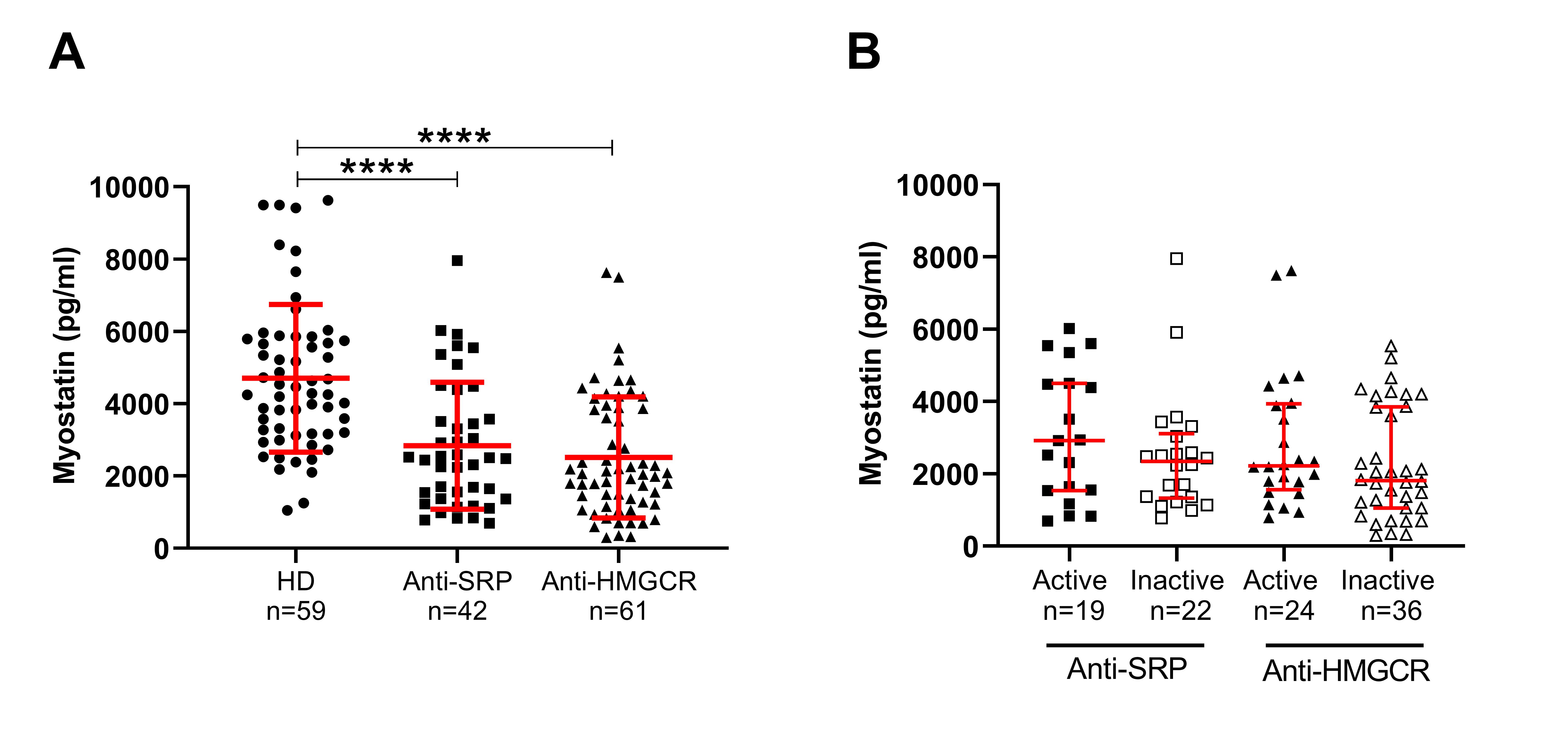

Supplement: Supplementary file 3 — Figure S3: Myostatin circulating levels in anti‐SRP and anti‐HMGCR IMNM patients Circulating myostatin levels in IMNM patients with anti‐SRP or anti‐HMGCR autoantibodies compared to controls (A) and in active or inactive patients (B). Patients with a PGA > 5 were considered active. ****p < 0.0001 [file NAN-49-0-s003.jpg]
